# Expression of miRNA in the Semitendinosus Muscle of Cattle Breeds with Varying Intramuscular Fat Deposition

**DOI:** 10.3390/genes16080969

**Published:** 2025-08-18

**Authors:** Anna Ciecierska, Abdolvahab Ebrahimpour Gorji, Alicja Majewska, Tomasz Sadkowski

**Affiliations:** Department of Physiological Sciences, Faculty of Veterinary Medicine, Warsaw University of Life Sciences—SGGW, Nowoursynowska 159, 02-776 Warsaw, Poland; anna_ciecierska@sggw.edu.pl (A.C.); abdolvahab_ebrahimpourgorji1@sggw.edu.pl (A.E.G.); alicja_majewska@sggw.edu.pl (A.M.)

**Keywords:** microRNA (miRNA), intramuscular fat (IMF), cattle, marbling, gene expression

## Abstract

**Background**: This study investigates the expression of microRNAs (miRNAs) in the semitendinosus muscle of cattle breeds with varying intramuscular fat (IMF) deposition to identify key miRNA regulators of beef marbling, utilizing Hereford (HER; higher IMF) and Holstein-Friesian (HF; moderate IMF) bulls, and Limousin (LIM; low IMF) bulls with lower IMF in the semitendinosus muscle. **Methods**: MicroRNA profiling used custom bovine microarrays and the Agilent software. The selected miRNAs, miR-34a, miR-149-5p, miR-208b, miR-499, miR-660, and miR-1343-5p, were chosen for validation using real-time PCR, confirming their differential expression. Target prediction utilized miRWalk, while functional and pathway analyses were conducted using the DAVID database to interpret biological relevance. **Results**: Microarray analysis identified 51 differentially expressed miRNAs. Among these, 24 exhibited consistent expression patterns in high-marbling breeds compared to the low-marbling LIM breed. Bioinformatic analysis of the 4941 predicted target genes of these 24 miRNAs revealed significant enrichment in pathways crucial for marbling, including the adipocytokine, AMPK, MAPK, and PI3K-Akt signaling pathways, as well as biological processes such as cell differentiation and lipid homeostasis. Notably, miR-34a and miR-149-5p emerged as significant regulators, with miR-34a targeting genes like *SIRT1*, *HMGA2*, *PTPN11*, *VEGFA*, *FGF1*, *FGF2*, and *BRAF*, and miR-149-5p influencing adipogenesis and lipid metabolism through its association with crucial KEGG pathways such as PI3K–Akt, MAPK, PPAR, TGF-β, cAMP, and Wnt signaling, all of which collectively influence adipocyte differentiation, lipid metabolism, cell cycle control, and angiogenesis. **Conclusions**: The findings underscore identified miRNAs’ possible coordinated regulatory role, particularly miR-34a and miR-149-5p, in the complex molecular mechanisms governing IMF deposition in cattle, providing potential targets for improving beef quality.

## 1. Introduction

The quality of livestock meat depends on many factors. One of the groups used to determine this parameter is sensory characteristics, including the associated intramuscular fat (IMF) content, which positively impacts flavor, juiciness, and tenderness of meat [1]. Marbled beef is characterized by the uneven distribution of IMF in skeletal muscle tissue, giving it a marble-like appearance.

Consecutive stages of adipose tissue development include preadipocyte proliferation and differentiation, adipocyte maturation, lipid filling, and metabolism, leading to increased IMF deposition and marbling development [2]. The postnatal fat accretion, including IMF deposition, depends on the expansion of adipose tissue volume, resulting from hyperplasia (characterized by an increase in adipocyte number) and hypertrophy (an increase in the size of existing adipocytes) [3,4,5]. The final stage of marbling development is lipid filling of the adipose cell body and adipocyte maintenance. The extent of IMF accumulation is heavily influenced by variations in genetic factors, which can enhance marbling due to inherited traits favoring adipogenesis. Multiple genes can affect this process by contributing to the following processes: glucose metabolism, energy homeostasis, fatty acid biosynthesis, fatty acid desaturation, lipid metabolism, fat cell development, beta oxidation, etc. This results in reduced or exaggerated IMF deposition. An example is the stearoyl-CoA desaturase (*SCD*) gene, which encodes an enzyme that catalyzes the conversion of saturated fatty acids into monounsaturated fatty acids, with genetic variations in *SCD* being associated with enhanced IMF deposition and improved marbling in cattle [6]. Marbling, or intramuscular fat deposition, is primarily studied in the longissimus dorsi but is also analyzed in other muscles like the gluteus medius and semitendinosus, depending on research goals, culinary traditions, and industry standards. Sadkowski et al. (2014) [7] also revealed significant differences in the expression of genes involved in IMF deposition in the semitendinosus muscle. However, little is known about the changes in miRNA expression and post-transcriptional gene expression during the development of the IMF in the semitendinosus muscle in cattle. Understanding the genetic mechanisms of IMF deposition is vital for improving beef quality and meeting market demands.

MicroRNAs (miRNAs) are small, noncoding RNAs that play essential roles in regulating gene expression at the post-transcriptional level. Most miRNAs are transcribed as independent transcripts, but approximately one-third are embedded within introns of protein-coding genes and processed following splicing of pre-messenger RNAs [8,9]. Although the specific biological roles of most miRNAs are still not fully known, functional characterization suggests that these small RNA molecules are involved in many processes of animal development [10,11], including adipogenesis [12]. The role of miRNAs in lipid metabolism was first reported in Drosophila, where the deletion of miRNA-14 increased the levels of triacylglycerol and diacylglycerol [12]. Additionally, miR-103, miR-143 [13], miR-17~92, miR-21 [14], and miR-204/211 have been reported to promote adipogenesis, while the miR-27 family inhibits this process [15]. miRNAs have also been differentially expressed in bovine adipose tissue, with the expression of miR-378 varying according to subcutaneous fat thickness [16]. This miRNA is also differentially expressed in murine adipocytes during differentiation [17], and its pro-adipogenic activity is possibly regulated by two tumor suppressor genes [18,19].

Some studies provide a comprehensive view of miRNA regulation in cattle fat metabolism. Jin et al. (2010) [16] established the role of miR-378 in subcutaneous fat, and miR-3432 significantly correlated with backfat thickness in cattle, suggesting its potential regulatory role in adipogenesis, the process of fat cell development. Wang et al. (2013) [20] highlighted distinct miRNA profiles between IMF and subcutaneous fat, with specific miRNAs like miR-143 and miR-145 being critical for IMF. Mir et al. (2020) [21] further linked DE miRNAs to IMF accumulation in muscle, offering potential targets for improving marbling. This analysis underscores the pivotal role of miRNAs in regulating fat metabolism and deposition in cattle, with significant implications for beef quality enhancement.

This study aimed to identify breed-independent, conserved molecular mechanisms of IMF deposition, based on IMF content variability in the semitendinosus muscle across different breeds (HER, HF, and LIM bulls). This comparative approach sought to minimize the influence of breed-specific genetic background and uncover key regulatory miRNAs, their downstream targets, and associated pathways, with the ultimate goal of improving marbling traits in diverse cattle populations, including dual-purpose breeds [22]. We hypothesized that variability in IMF content among these breeds may be linked to specific miRNA expression patterns in the semitendinosus muscle. These miRNAs could influence adipocyte biology and lipid handling by modulating genes and pathways involved in adipogenesis, energy metabolism, and tissue remodeling. Elucidating such post-transcriptional mechanisms may shed light on conserved regulatory elements contributing to fat deposition in muscle, beyond breed-specific effects.

## 2. Materials and Methods

### 2.1. Ethics Statement

This study complies with national and institutional guidelines of the use of animals in research according to the Polish Legal Act of 21 January 2005. Since sample collection was performed during routine slaughter and no additional harmful or painful procedures for the animals were applied, this study did not require formal ethical approval.

### 2.2. Animals and Muscle Samples Collection

The semitendinosus muscle was collected from 15-month-old HER (high IMF; beef cattle), HF (moderate IMF; dairy cattle), and LIM (low IMF; beef cattle) bulls differing in IMF deposition (*n* = 15 per breed) [7]. Starting from the age of 2–3 months, the bulls were housed in a farm of the Institute of Genetics and Animal Breeding of the Polish Academy of Sciences in Jastrzębiec (IGAB PAN), in a loose barn, until slaughter. The animals were fed ad libitum a total mixed ration (TMR) consisting of corn silage (75%), concentrates (20%), and hay (5%) and had free access to water. At 15 months, all bulls were slaughtered at the abattoir of IGAB PAN after 24 h of fasting. Muscle samples for total RNA isolation, measuring 0.5 × 0.5 cm^2^, were collected immediately after slaughter. Visible connective tissue was removed, and the cleaned samples were immersed in liquid nitrogen. Subsequently, the samples were stored at −80 °C until further analysis. Then, the carcasses were chilled for 24 h at 4 °C and then dissected into lean, fat, and bone [7,23,24]. In previous research, Soxhlet analysis revealed significant differences in intramuscular fat in the semitendinosus muscle between HER (1.10%), HF (0.81%), and LIM (0.53%). CVS marbling assessment confirmed this pattern (HER, 3.23 ± 0.23%; HF, 2.61 ± 0.19%; and LIM, 1.57 ± 0.09%), and dry matter and protein content did not differ significantly between breeds [7].

### 2.3. RNA Isolation and Validation

The semitendinosus muscle samples from individuals with IMF content close to the breed-specific average (based on Soxhlet analysis) were selected. Total RNA, including miRNA, was extracted using the miRNeasy Mini Kit (Qiagen, Ermentown, MD, USA) according to the manufacturer’s instructions and finally eluted with RNase-free water. The RNase inhibitor (Sigma-Aldrich, Louis, MO, USA) was added to each sample. RNA concentration and purity were assessed using a NanoDrop spectrophotometer (Nanodrop Technologies, Wilmington, DE, USA) by measuring absorbance at 260, 280, and 230 nm; optimal purity was confirmed by A260/A280 and A260/A230 ratios of ~2.0 and ≥1.8, respectively. Quality and integrity were measured by a Bioanalyzer 2100 (Agilent Technologies, Santa Clara, CA, USA). Samples with RIN ≥ 8 (RNA Integrity Number) were included in the subsequent analysis.

### 2.4. miRNA Microarray Analysis

The analysis of miRNA profiling was performed using custom bovine miRNA microarrays (8 × 60 K) (Agilent Technologies, Santa Clara, CA, USA). The Agilent Array platform was used to design the microarrays (GPL19028, Agilent-049625, Bos taurus_miRNA, Santa Clara, CA, USA). As recommended by the manufacturer, 100 ng of the total RNA sample (*n* = 4, each in two technical repetitions) was labeled with the fluorescent dye Cyanine 3-pCp (Cy3) (Agilent Technologies, Santa Clara, CA, USA) and hybridized to probes on the bovine miRNA microarray. miRNA labeling, hybridization, and washing were performed following the manufacturer’s protocol, version 2.3, and the miRNA Microarray System with miRNA Complete Labeling and Hyb Kit (Agilent Technologies, Santa Clara, CA, USA). Images of hybridized microarrays were acquired with an Agilent Microarray Scanner (G2565BA; Agilent Technologies, Santa Clara, CA, USA), and features were extracted using the Agilent Feature Extraction (Agilent Technologies, Santa Clara, CA, USA) image analysis tool, version A.9.5.3.1, with default protocols and settings. The statistical analysis was performed using the GeneSpring 13 software (Agilent Technologies, Santa Clara, CA, USA). The statistical significance was evaluated using one-way analysis of variance (ANOVA) [25] with Benjamini–Hochberg multiple testing correction adjustment (False Discovery Rate; FDR). FDR ≤ 0.05 was considered statistically significant. Fold change (FC) ≥ 1.0 was used.

The data obtained in the microarray experiment were deposited in the National Center for Biotechnology Information (NCBI) Gene Expression Omnibus database (GEO) with accession number GSE95398.

### 2.5. Real-Time qPCR Procedure

The expression of selected miRNAs was measured using real-time polymerase chain reaction (qPCR) to verify microarray results. First-strand cDNA synthesis was performed using the miRCURY LNA™ Universal RT cDNA Synthesis Kit II (Exiqon, Denmark). UniSp6 Spike-in was used for quality control. All analyses were performed using a SYBR^®^ Green master mix, Universal RT (Exiqon, Vedbaek, Denmark), according to the manufacturer’s protocol, the procedure described earlier by Sadkowski et al. (2018) [24]. All reactions were performed in triplicate (*n* = 15 per breed). The primers are listed in Table 1. The amplification was performed in a Stratagene Mx3005P thermal cycler (Agilent Technologies, Santa Clara, CA, USA) in FrameStar 96 PCR plates (BioLab Innovative Research Technologies, Poland). The U6 snRNA gene was used as a reference. The expression of miRNA was determined using the ΔΔCt method [26].

### 2.6. Target Gene Prediction Protocol

To elucidate miRNA-regulated genes, miRWalk (http://mirwalk.umm.uni-heidelberg.de/, accessed on 15 May 2025), an advanced miRNA target prediction platform, was employed. The analysis incorporated multiple filtering criteria to optimize prediction precision, including free energy (ΔG, kcal/mol) of the miRNA-mRNA duplex, where more negative values denote enhanced binding stability; binding probability, with values approaching 1 indicating higher confidence; and seed scores, which assess the robustness of seed region interactions between miRNAs and target mRNAs. Genes exhibiting regulatory interactions were validated using the miRWalk algorithmic scoring. High-confidence targets were selected, enabling comprehensive functional and pathway analyses aligned with the study’s objectives.

### 2.7. Functional Analysis

Pathway and Gene Ontology (GO) enrichment analyses were conducted using the DAVID database (https://davidbioinformatics.nih.gov/, accessed on 15 May 2025). The study focused on the biological process in GO categories. Enriched GO terms and signaling pathways were identified using Fisher’s exact test with a significance threshold of *p*-value ≤ 0.05. Statistically significant results were visualized using an online tool (http://www.bioinformatics.com.cn/srplot, accessed on 15 May 2025) for further interpretation.

### 2.8. Quantitative Trait Loci Annotation of Identified Genes

First, the QTL annotation file, QTLdb_cattleARS_UCD2.gff, was downloaded from the Animal Quantitative Trait Loci (QTL) Database (Animal QTLdb) at the following URL: https://www.animalgenome.org/cgi-bin/QTLdb/BT/index, accessed on 15 July 2025. This file provides genomic coordinates and trait information for all known bovine QTLs.

Next, the biomaRt package in R (2024.12.0 Build 467) was used to query the Ensembl database for gene annotations. A custom R script was developed to identify all genes whose genomic coordinates overlapped with the QTL regions defined in the QTLdb_cattleARS_UCD2.gff file. Specifically, for each QTL, the script extracted the start and end coordinates and then used the getBM() function from the biomaRt package to retrieve all genes located within that specific genomic interval. The Ensembl dataset “btaurus_gene_ensembl” was utilized, corresponding to the Bos taurus ARS-UCD1.2 genome assembly.

### 2.9. Statistical Analysis

Initial qPCR data analysis was performed using GenEX 6.0 (MultiD Analyses AB, Mölndal, Sweden). Furthermore, the data were analyzed using Prism 5.0 (GraphPad Software, San Diego, CA, USA), where ANOVA and Tukey’s multiple range test were applied. Results with a *p*-value ≤ 0.05 were considered statistically significant. The data are shown as means +/− standard error of the mean (SEM).

## 3. Results

### 3.1. Microarray Analysis

**Figure 1:**
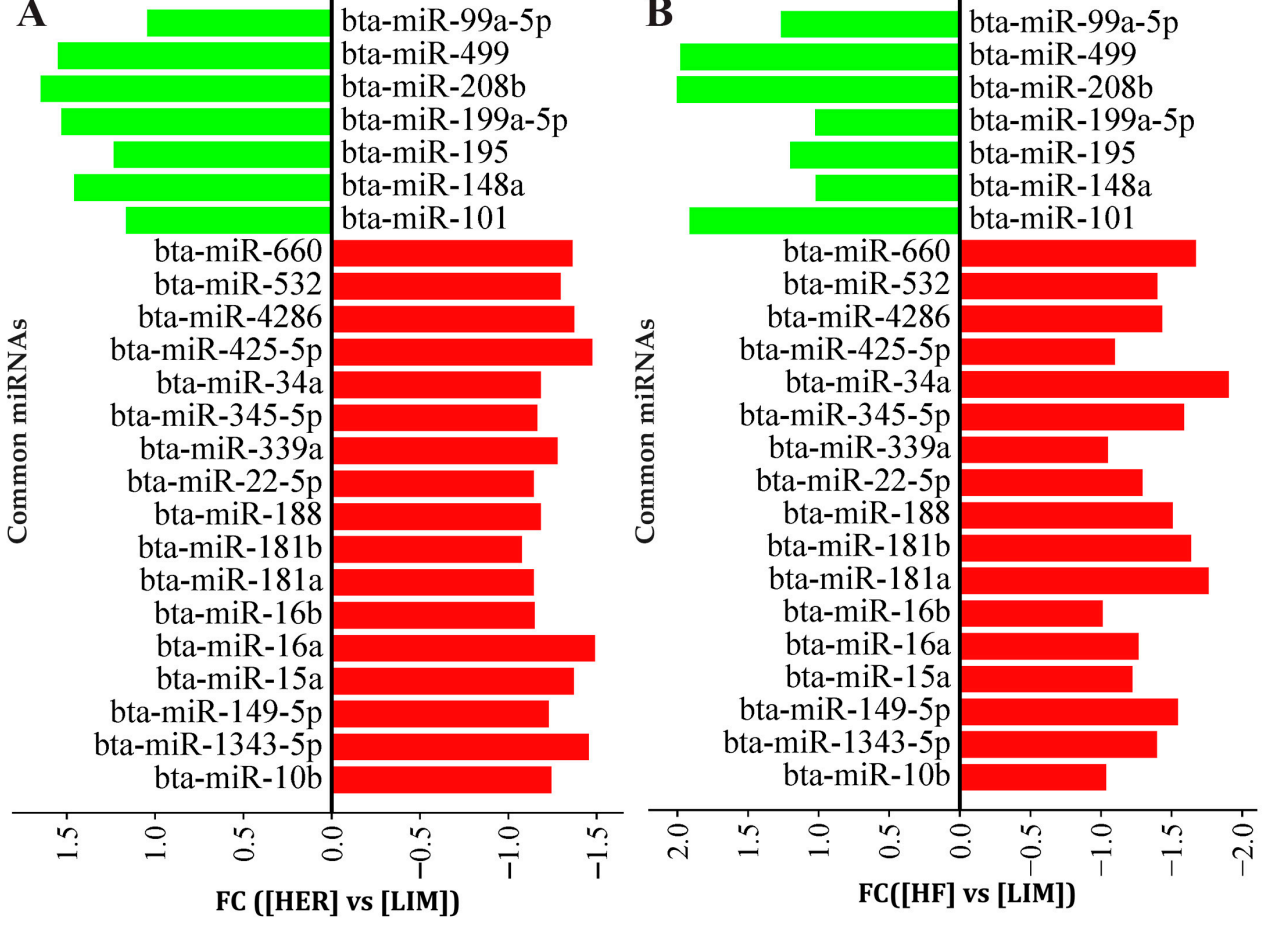
The expression of common miRNAs for (**A**) HER/LIM and (**B**) HF/LIM comparisons. Upregulation and downregulation are shown in green and red colors, respectively. False discovery rate (FDR) ≤ 0.05; fold change (FC) ≥ 1.0; *n* = 4 per breed. HER—Hereford; HF—Holstein-Friesian; LIM—Limousin.

Analysis of miRNA expression showed statistically significant differences in 51 miRNA molecules in the semitendinosus muscle of the examined breeds. Of these, 24 miRNAs have the up- or downregulation of expression in both higher-IMF breeds (HER and HF) vs. the lower-IMF (LIM) breed. In these 24 miRNAs, 7 miRNAs were upregulated, and 17 miRNAs were downregulated (Figure 1, Table S1).

### 3.2. Real-Time qPCR Validation

**Figure 2:**
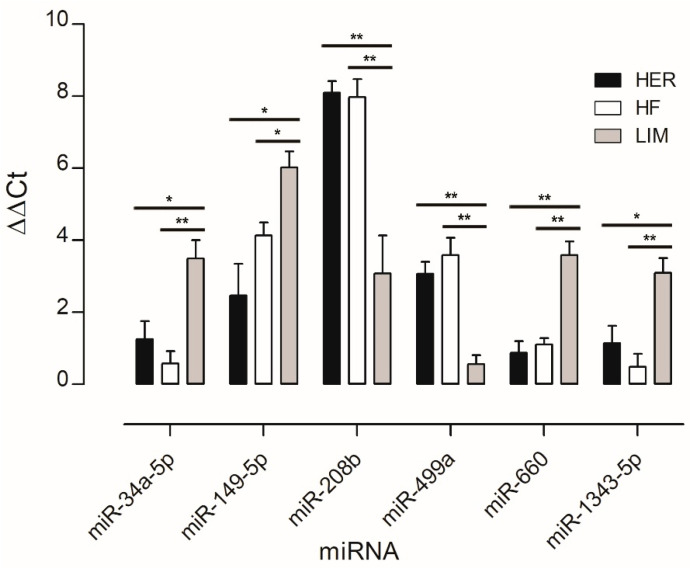
qPCR verification of the microarray results. Results are presented as mean ± standard error (SEM) and denoted as statistically significant * for *p* < 0.05 and ** for *p* < 0.01; *n* = 15 per breed. HER—Hereford; HF—Holstein-Friesian; LIM—Limousin.

miRNAs associated with adipose tissue development and lipid metabolism, and those not previously linked to marbling, were selected for validation by qPCR to confirm differences between the breeds studied. The analysis showed lower expression levels of four miRNAs (miR-34a-5p, miR-149-5p, miR-660, and miR-1343-5p) and higher expression levels of two miRNAs (miR-208b and miR-499a) in both HER/HF bulls relative to LIM (Figure 2). These confirm the results obtained by the microarray method (Figure 1; Table S1).

### 3.3. Target Genes Predicted for Identified miRNAs

**Figure 3:**
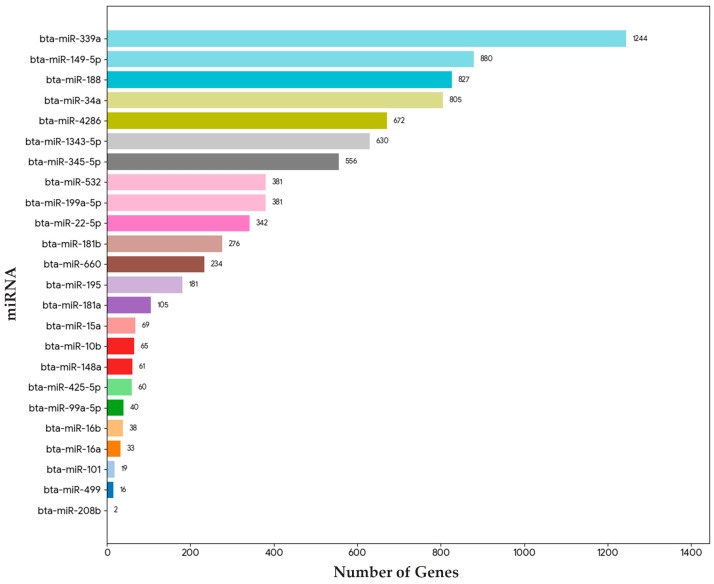
Number of unique genes associated with identified miRNAs.

Our comparative analysis identified 24 miRNAs exhibiting similar expression change in the semitendinosus muscle of bulls with better IMF deposition. Target genes regulated by these miRNAs were predicted using miRWalk, yielding 7917 genes, of which 4941 were unique genes regulated by the identified miRNAs (Tables S2 and S3). The miRNAs with the highest number of associated genes were miR-339a (1244 genes), miR-149-5p (880 genes), and miR-188 (827 genes), indicating their significant regulatory roles across a wide range of target genes. In contrast, miR-208b and miR-499a exhibited the lowest number of associated unique genes (2 and 16, respectively), suggesting their more limited or specialized regulatory function (Figure 3).

### 3.4. KEGG Pathway GO Enrichment Analysis

**Figure 4:**
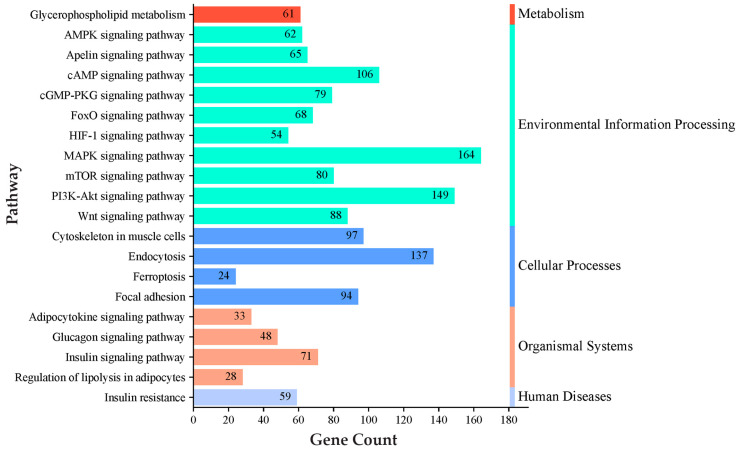
KEGG pathway analysis of predicted target genes for differentially expressed miRNAs. A complete list of the identified pathways is provided in Table S4.

**Figure 5:**
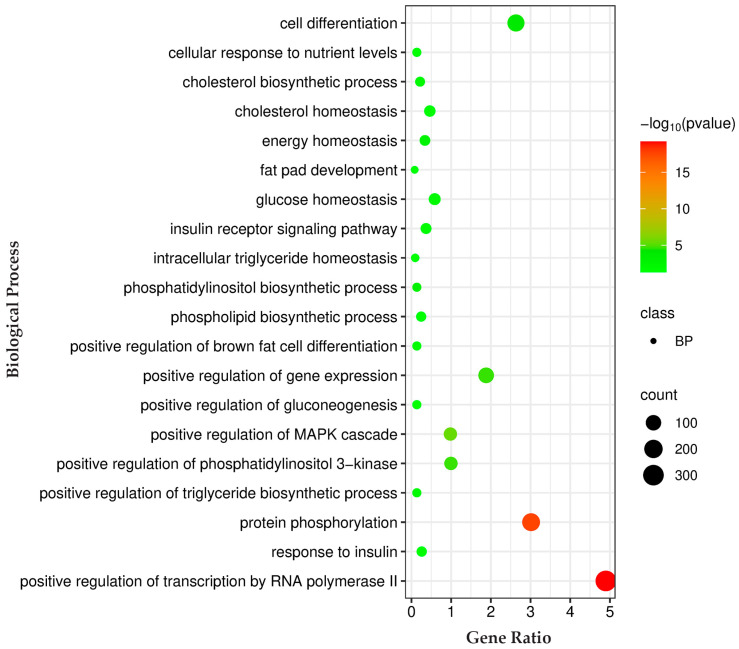
Biological process enrichment analysis of predicted target genes for differentially expressed miRNAs. Gene Ratio—fraction of input genes linked to a given GO term. Bubble size represents the number of genes involved in each term, while color indicates statistical significance (−log10(*p*-value)), with red denoting the most significant biological process. A complete list of the identified biological processes is provided in Table S5.

Pathway analysis for genes regulated by these miRNAs was conducted using the KEGG databases. The KEGG pathway analysis prioritized key pathways based on the number of genes associated with them. Among these pathways, we selected the top enriched pathways related to IMF deposition, such as the adipocytokine signaling pathway, the AMPK signaling pathway, the cAMP signaling pathway, the glucagon signaling pathway, glycerophospholipid metabolism, the insulin signaling pathway, the MAPK signaling pathway, the mTOR signaling pathway, the PI3K-Akt signaling pathway, and regulation of lipolysis in adipocytes (Figure 4; Table S4). Biological process analysis of the genes regulated by identified miRNAs showed enrichment for cell differentiation, cholesterol biosynthetic process, cholesterol homeostasis, energy homeostasis, fat pad development, glucose homeostasis, insulin receptor signaling pathway, intracellular triglyceride homeostasis, the phosphatidylinositol biosynthetic process, and the phospholipid biosynthetic process (Figure 5; Table S5).

### 3.5. miRNA–Gene–QTL

The analysis of the miRNA–gene–QTL interactions in cattle revealed significant associations between specific miRNAs and genes linked to marbling score and intramuscular fat traits. Utilizing the QTLdb_cattleARS_UCD2.gff file from the Animal QTLdb and gene annotations from the Ensembl database via the biomaRt package (2024.12.0 Build 467), a custom R script identified overlapping genomic coordinates to map 369 unique miRNA–gene–QTL interactions across 101 records. Key miRNAs, including bta-miR-34a, bta-miR-149-5p, bta-miR-188, bta-miR-660, and bta-miR-1343-5p were frequently associated with genes such as *ABL1*, *AK4*, *BCAT1*, and *NR6A1*, predominantly on chromosomes 1, 2, 3, 5, 6, 7, 10, 11, 13, 16, 17, 18, 20, 21, 22, 23, 24, 25, 26, 27, 28, and 29. The marbling score trait dominated the dataset, with 97 records, while IMF appeared in 4 instances. Interestingly, genes like *FRAS1* and *RAB30* showed multiple QTL hits, suggesting complex regulatory networks (Table S3).

**Figure 6:**
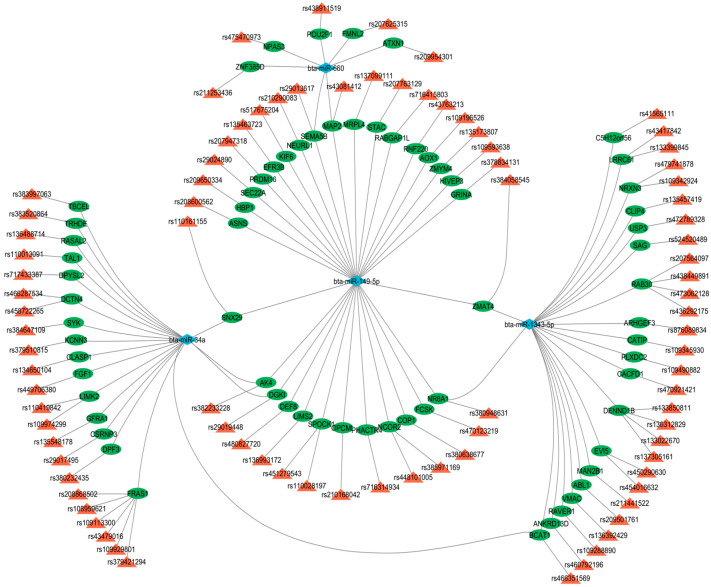
The network visualizes the complex regulatory relationships between miRNAs (blue diamond), confirmed by qPCR, and their target genes (green circle nodes), which are located within QTLs (represented by red triangle nodes) for the marbling score and IMF trait.

Figure 6 provides a visual representation of these complex regulatory interactions. The network highlights the intricate relationships, showing how specific miRNAs can regulate multiple target genes associated with the marbling score and IMF. The centrality of certain miRNAs, notably, bta-miR-34a, miR-149-5p, and bta-miR-1343-5p, is evident, as they form connections with numerous genes, suggesting a major role in the overall genetic architecture of this economically important trait.

## 4. Discussion

Intramuscular fat content, commonly referred to as marbling, is a critical determinant of meat quality, influencing sensory attributes such as tenderness, juiciness, and flavor in beef. miRNAs, small non-coding RNAs, serve as pivotal post-transcriptional regulators of gene expression, modulating key pathways involved in adipogenesis, lipid metabolism, and fatty acid biosynthesis that drive IMF deposition. This study aimed to identify miRNAs associated with better IMF deposition and quality in the semitendinosus muscle of HER and HF bulls, related to LIM cattle, with the goal of uncovering breed-independent mechanisms of IMF deposition.

Microarray analysis identified 24 differentially expressed miRNAs showing consistent patterns across both HER/HF cattle (Figure 1; Table S1). qPCR analysis confirmed their differential expression, consistent with the microarray analysis results (Figure 2). Bioinformatic analysis of their 4941 predicted target genes (Figure 3; Table S2) revealed significant enrichment in pathways (Table S3) crucial for marbling, including the adipocytokine, AMPK, MAPK, and PI3K-Akt signaling pathways (Figure 5), as well as biological processes (Table S4) like cell differentiation and lipid homeostasis (Figure 6). This highlights a coordinated regulatory role for these miRNAs, particularly miR-34a and miR-149-5p, in the complex molecular mechanisms governing IMF deposition in cattle.

### 4.1. miR-34a and miR-149-5p as Key Regulators of IMF Deposition

Among the identified miRNAs, miR-34a emerged as a key regulator of IMF deposition by targeting numerous genes involved in adipogenesis, lipid metabolism, cell cycle control, and angiogenesis [27,28]. Notably, miR-34a suppresses sirtuin 1 (*SIRT1*) by binding to its 3′UTR, thereby influencing TP53-dependent apoptosis and cell cycle arrest, processes linked to adipogenesis in muscle tissue [29,30,31]. It also represses high-mobility group AT-hook 2 (*HMGA2*), limiting the proliferation and differentiation of adipogenic precursors [28,32,33], and targets protein tyrosine phosphatase non-receptor type 11 (*PTPN11*), potentially affecting growth factor signaling and adipose tissue development [34,35]. Vascularization, essential for IMF accumulation, may also be impaired by miR-34a via repression of vascular endothelial growth factor A (*VEGFA*), fibroblast growth factor 1 (*FGF1*), and fibroblast growth factor 2 (*FGF2*), all critical for angiogenesis and tissue remodeling [28,36]. Additionally, miR-34a is predicted to inhibit Acyl-CoA synthetase long-chain family member 4 (*ACSL4*), thereby impacting lipid metabolism and further limiting IMF deposition [37]. By simultaneously modulating the adipogenesis, angiogenesis, and metabolic processes, miR-34a acts as a central regulatory node in muscle remodeling and fat deposition [28,37,38]. Its role in targeting the B-Raf proto-oncogene serine/threonine kinase (*BRAF*) within the MAPK pathway further highlights its broad impact on cell growth and differentiation. Similar functions observed for miR-34b in lipid synthesis inhibition reinforce the broader regulatory role of the miR-34 family in IMF development [39]. In our study, the decreased expression of miR-34a in high- and moderate-IMF breeds (HER/HF) supports its proposed inhibitory role in fat deposition, as lower miR-34a levels are associated with enhanced IMF accumulation.

Beyond miR-34a, miR-149-5p also appears to be a key regulator of bovine IMF development and quality. Functional assays have shown that miR-149-5p mimics significantly reduce lipid droplet accumulation in bovine adipocytes, indicating a strong inhibitory effect on adipogenesis [40]. Pathway enrichment analyses further associate miR-149-5p with several key KEGG pathways involved in lipid metabolism and fat deposition, including PI3K–Akt, MAPK, PPAR, TGF-β, cAMP, and Wnt signaling [40,41]. Similar results observed in porcine models support the conserved role of miR-149-5p as a negative regulator of fat accumulation across species [42], suggesting its relevance in controlling adipocyte differentiation and marbling in cattle [21]. Additionally, the study by Wang et al. (2020) [37] demonstrated that miR-34a suppresses adipogenesis in porcine intramuscular adipocytes by targeting and downregulating ACSL4, a gene essential for long-chain fatty acid activation and lipid metabolism. The regulatory actions of both miR-34a and miR-149-5p contribute to the inhibition of lipid accumulation and adipocyte differentiation, thereby modulating IMF deposition and muscle remodeling processes in pigs, and, as evidenced by our results, in cattle as well. Notably, both miRNAs were found to be downregulated in our study, supporting their potential roles as negative regulators of IMF development.

### 4.2. Regulatory Roles of Other miRNAs in Adipogenesis and Muscle Remodeling

Other validated miRNAs, such as miR-208b, miR-499a, miR-660, and miR-1343-5p, can directly or indirectly influence adipogenesis, lipid metabolism, lipid storage, and muscle tissue remodeling, which are critical for IMF development in skeletal muscles like the longissimus dorsi and semitendinosus. In rabbits, overexpression of miR-208b increased peroxisome proliferator-activated receptor gamma (*PPARG*) and fatty acid-binding protein 4 (*FABP4*) expression and lipid droplet accumulation [43], suggesting a promotive role in preadipocyte differentiation. That study also predicted casein kinase 2 alpha 2 (*CSNK2A2*) as a direct target of miR-208b [43]. In contrast, miR-499 suppresses adipogenesis in pigs by targeting PR/SET domain 16 (*PRDM16*), reducing lipid accumulation and adipocyte differentiation, and finally limiting IMF deposition [44]. The impact of this miRNA on the stabilization of muscle fiber types during tissue remodeling, along with IMF deposition, may potentially disturb extracellular matrix (ECM) reorganization and angiogenesis, which are necessary for proper adipocyte integration into muscle tissue [43,45,46]. Although the role of miR-660 in bovine IMF remains unclear, studies on Nelore cattle suggest it may influence muscle remodeling via TGF-β signaling and ECM regulation [47]. Another molecule that has been validated, and whose involvement in IMF deposition and lipid metabolism has not been confirmed so far, miR-1343, is associated with autophagy and lipid turnover [48] and may act in concert with miR-34a, which directly affects SIRT1, involved in the process of autophagy/lipophagy, and thus controls lipid droplet catabolism [47]. The involvement of miR-1343 in IMF deposition is further supported by its identification as a backfat-associated miRNA in Large White and Chinese Meishan pigs [49]. In summary, miR-34a, miR-149-5p, miR-208b, miR-660, and miR-1343 may promote IMF deposition through diverse mechanisms, while miR-499 appears to favor muscle development at the expense of IMF accumulation, two processes that occur concurrently.

Additionally, several other miRNAs identified in our transcriptome analysis may influence IMF development. miR-339a is linked to insulin signaling and lipid metabolism [50,51], while miR-148a, miR-143, and miR-10b are associated with adipocyte differentiation and adipogenesis [40,52,53]. miR-181a and miR-196a regulate myogenic cell proliferation and differentiation [54], with miR-181a also associated with fat necrosis [55]. miR-199a-5p modulates lipid metabolism during preadipocyte differentiation and bovine adipocytes [40,55,56]. Other miRNAs, such as miR-16a/b, miR-22-5p, miR-4286, miR-188, and miR-345-5p, are involved in regulatory networks affecting adipogenesis, lipid metabolism, and mitochondrial function [21,57]. Although direct evidence is limited, their roles in ceRNA networks suggest a contribution to IMF regulation [41].

Our study identified several miRNAs associated with key candidate genes regulating IMF deposition in cattle semitendinosus muscle, as reported by Sadkowski et al. (2014) [7]. miR-149-5p represses adipogenic markers linked to agouti signaling protein (*ASIP*) and estrogen receptor 1 (*ESR1*), while solute carrier family 29 member 2 (*SLC29A2*), also associated with miR-149-5p, regulates preadipocyte differentiation [40,58]. *ESR1*, connected to miR-16a/b, is part of the marbling-related gene profile and influences adipocyte development [59]. Bioinformatic analyses suggest that these miRNAs target gene sets involved in lipid metabolism and muscle development, shaping the balance between adipogenesis and myogenesis [57,60]. Although miRNA–mRNA interactions involving tripartite motif containing 32 (*TRIM32*), SET domain bifurcated histone lysine methyltransferase 1 (*SETDB1*), and *SLC29A2* require further validation, their presence in ceRNA networks highlights the role of miRNAs in post-transcriptional regulation of IMF deposition [21].

### 4.3. Integrative Analysis of miRNAs, Target Genes, and Trait-Associated QTLs

Analysis of the dataset reveals critical miRNA–gene–QTL associations significantly linked to marbling and IMF deposition in cattle traits that are fundamental to meat quality, particularly in influencing tenderness, flavor, and juiciness. Notably, among the top-ranked associations, bta-miR-34a, targeting *FGF1* (linked to QTL rs449706380), and bta-miR-195, targeting *FNDC3A* (associated with QTLs rs109083743, rs210708771, rs209486540, rs41566737, rs136495094, rs209664284, rs211286798, rs209584346, rs134522885, rs110981446, rs133596146, rs135266639, and rs41574267) [61], demonstrate high statistical significance, suggesting potent regulatory roles in lipid metabolism.

*FGF1*, a fibroblast growth factor, is well established in promoting adipocyte differentiation, a key mechanism underpinning IMF accumulation, as corroborated by the existing literature regarding its role in lipid deposition [27,62]. Similarly, *FNDC3A*, which appears in ten instances within the dataset, is involved in extracellular matrix organization, potentially contributing to the stabilization of muscle fat tissue interactions that are crucial for marbling development [63,64].

bta-miR-199a-5p targeting *SCD* is connected to four QTLs (rs41255691, rs41255692, rs41255693, and rs383175036) [33]. This interaction is particularly significant given SCD’s central role in fatty acid biosynthesis; it catalyzes the formation of monounsaturated fatty acids, thereby directly enhancing IMF content and marbling quality [63,65]. The recurrent targeting of SCD by multiple miRNAs, including bta-miR-339a and bta-miR-4286, further emphasizes its pivotal position in lipid metabolic regulation.

Moreover, bta-miR-199a-5p also targets *MTMR2*, associated with QTL rs379513186 [61], which is implicated in lipid signaling, thereby reinforcing its role in IMF development. Other notable associations include bta-miR-22-5p, targeting *SLC20A2* (QTL rs207664433), and bta-miR-532, targeting *VOPP1* (QTLs rs443087147 and rs210538473) [61]. SLC20A2 plays a role in phosphate transport, potentially influencing energy dynamics in adipocytes [66], while *VOPP1* may regulate vesicular trafficking mechanisms related to lipid storage [67].

Additionally, bta-miR-181b targets *SH2D3A* on chromosome 7 (QTLs rs43284251 and rs41656917) [68], a gene associated with signaling pathways relevant to fat deposition. bta-miR-532 and bta-miR-149-5p target *KALRN* (QTL rs439053804, chromosome 1) and *RABGAP1L* (QTL rs716415803, chromosome 16) [61], respectively. These genes are involved in cytoskeletal dynamics and vesicle trafficking, which may indirectly facilitate IMF accumulation by supporting intracellular lipid transport [40,53]. Furthermore, bta-miR-34a targets *AK4* (QTL rs382233228) [61], suggesting a role in energy metabolism during adipogenesis (Figure 4).

Collectively, these miRNAs fine-tune a network of post-transcriptional regulation impacting key biological processes, including lipid biosynthesis (*SCD*; *FGF1*), cellular structure and integrity (*FNDC3A*; *KALRN*), and signaling pathways (*MTMR2*; *SH2D3A*) [69]. The identified QTLs, particularly those linked to *FGF1* and *FNDC3A*, delineate genomic loci with strong effects on marbling and IMF traits, supported by low p-values and the corroborating literature.

This study’s limitations include its focus on a single muscle, the semitendinosus, which may not represent overall IMF deposition across the entire carcass. The analysis primarily relies on predicted miRNA–target gene interactions, requiring further functional validation to confirm direct regulatory relationships. Additionally, the study’s scope does not include an in-depth mechanistic elucidation of all identified miRNAs. The study provides a foundation for further in-depth research.

## 5. Conclusions

**Figure 7:**
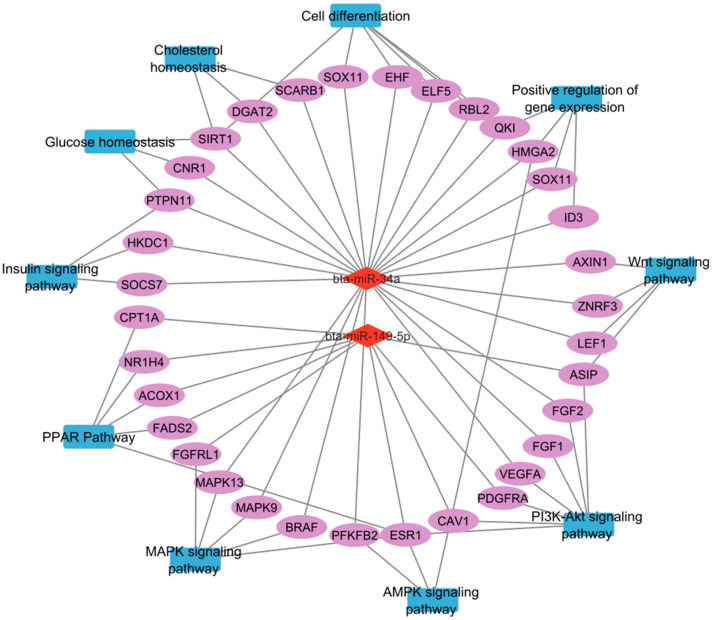
Proposed regulatory network of key miRNAs and their target genes involved in pathways critical for adipogenesis, lipid metabolism, cell differentiation, and angiogenesis, ultimately impacting intramuscular fat deposition and quality in cattle. In this diagram, red diamonds represent miRNAs, ovals represent genes, and blue rectangles represent pathways.

This study identified several miRNAs differentially expressed in the semitendinosus muscle of cattle breeds with varying IMF content, revealing breed-independent mechanisms of IMF deposition. The results highlight the significant role of miRNAs, especially miR-34a and miR-149-5p (Figure 7), in regulating key biological processes and signaling pathways critical for IMF deposition, marbling development, and skeletal muscle tissue remodeling. These insights expand our understanding of molecular determinants of beef quality and point to potential targets for genetic improvement and dietary modulation.

To apply these findings in breeding practice, key regulatory interactions should first be functionally validated and genetic markers confirmed in large populations. Integrating multi-omics data can improve selection accuracy by capturing the complexity of IMF regulation. Genomic selection, supported by validated SNP panels, along with tailored feeding strategies and targeted crossbreeding, may help enhance marbling in a breed-specific and consumer-oriented manner.

## Figures and Tables

**Table 1:** miRNA primers used for qPCR (Exiqon, Vedbaek, Denmark).

Primer	Target Sequence	Accession
bta-miR-34a LNA™ PCR primer set, UniRT	UGGCAGUGUCUUAGCUGGUUGU	MIMAT0004340
bta-miR-149-5p LNA™ PCR primer set, UniRT	UCUGGCUCCGUGUCUUCACUCCC	MIMAT0024570
bta-miR-208b LNA™ PCR primer set, UniRT	AUAAGACGAACAAAAGGUUUGU	MIMAT0009262
bta-miR-499 LNA™ PCR primer set, UniRT	UUAAGACUUGCAGUGAUGUUU	MIMAT0003536
bta-miR-660 LNA™ PCR primer set, UniRT	UACCCAUUGCAUAUCGGAGCUG	MIMAT0004344
bta-miR-1343-5p LNA™ PCR primer set, UniRT	UGGGGAGCGGCCCCCGGGCGGG	MIMAT0011839
U6 snRNA LNA™ PCR primer set, UniRT (reference)	GUGCUCGCUUCGGCAGCACAUAU ACUAAAAUUGGAACGAUACAGAG AAGAUUAGCAUGGCCCCUGCGCA AGGAUGACACGCAAAUUCGUGAA GCGUUCCAUAUUUUU	#203907

## Data Availability

The data obtained in the microarray experiment were deposited in the National Center for Biotechnology Information (NCBI) Gene Expression Omnibus database (GEO) with accession number GSE95398.

## Supplementary Materials

The following supporting information can be downloaded at: https://www.mdpi.com/article/10.3390/genes16080969/s1, Table S1: MiRNAs with similar expression change in higher-marbled HER/HF in comparison to lower-marbled LIM bulls; Table S2: Predicted target genes associated with identified miRNAs; Table S3: Integrated mapping of miRNAs, their predicted target genes, and associated QTLs for intramuscular fat and related traits in cattle; Table S4: KEGG pathway analysis of predicted target genes; Table S5: Biological process enrichment analysis of predicted target genes.

## Abbreviations

The following abbreviations are used in this manuscript:

AMPK	AMP-activated protein kinase
ANOVA	Analysis of variance
ASIP	Agouti signaling protein
BRAF	B-Raf proto-oncogene, serine/threonine kinase
CDNA	Complementary deoxyribonucleic acid
CY3	Cyanine 3-pCp
ECM	Extracellular matrix
ESR1	Estrogen receptor 1
EU	European Union
FABP4	Fatty acid-binding protein 4
FC	Fold change
FDR	False Discovery Rate
FGF1	Fibroblast growth factor 1
FGF2	Fibroblast growth factor 2
FUS1	Fused in sarcoma
GEO	Gene Expression Omnibus
GO	Gene Ontology
HER	Hereford
HF	Holstein-Friesian
HMGA2	High-mobility group AT-hook 2
IMF	Intramuscular fat
LIM	Limousin
MAPK	Mitogen-activated protein kinase
miRNAs	microRNAs
NCBI	National Center for Biotechnology Information
PI3K-AKT	Phosphoinositide 3-kinase-Akt
PPARG	Peroxisome proliferator-activated receptor gamma
PRDM16	PR/SET domain 16
PTPN11	Protein tyrosine phosphatase non-receptor type 11
QPCR	Quantitative Polymerase Chain Reaction
QTL	Quantitative Trait Loci
RIN	RNA Integrity Number
SCD	Stearoyl-CoA desaturase
SEM	Standard error of the mean
SETDB1	SET domain bifurcated histone lysine methyltransferase 1
SIRT1	Sirtuin 1
SLC29A2	Solute carrier family 29 member 2
SNR-NA	Small nucleolar RNA
SUFU	Suppressor of fused homolog
SYBR	SYBR Green
TMR	Total mixed ration
TRIM32	Tripartite motif containing 32
VEGFA	Vascular endothelial growth factor A
