# Supplementary material for: Expression of miRNA in the Semitendinosus Muscle of Cattle Breeds with Varying Intramuscular Fat Deposition
Source: Genes (Basel). 2025 Aug 18;16(8):969. doi: 10.3390/genes16080969 (PMC12385881; doi:10.3390/genes16080969)
Supplement: Supplementary file 1 [file genes-16-00969-s001.zip › Table S1.pdf]

Table 1. MiRNAs with similar expression change in higher-marbled HER/HF in comparison to lower-marbled LIM bulls.

No.	Systematic name	FC ([HER] vs [LIM])	Regulation ([HER] vs [LIM])	FC ([HF] vs [LIM])	Regulation ([HF] vs [LIM])	Active sequence
1	bta-miR-1343-5p	-1.45	down	-1.3955224	down	CCCGCCCGGGG
2	bta-miR-34a	-1.18	down	-1.9030912	down	ACAACCAGCTAAGACACTGC
3	bta-miR-660	-1.36	down	-1.6706836	down	CAGCTCCGATATGCAA
4	bta-miR-10b	-1.24	down	-1.0338962	down	CACAAATTCGGTTCTACAGGG
5	bta-miR-149-5p	-1.23	down	-1.5430462	down	GGGAGTGAAGACACGGAG
6	bta-miR-15a	-1.37	down	-1.2217649	down	ACAAACCATTATGTGCTGC
7	bta-miR-16a	-1.49	down	-1.2634065	down	CACCAATATTTACGTGCT
8	bta-miR-16b	-1.15	down	-1.011718	down	GCCAATATTTACGTGCTG
9	bta-miR-181a	-1.1432683	down	-1.7622936	down	AACTCACCGACAGCG
10	bta-miR-181b	-1.075301	down	-1.637034	down	AACCCACCGACAGC
11	bta-miR-188	-1.1841471	down	-1.5071055	down	ACCCTCCACCATGCA
12	bta-miR-22-5p	-1.1445036	down	-1.292388	down	TAAAGCTTGCCACTGAAG
13	bta-miR-339a	-1.2790455	down	-1.0469768	down	GTGAGCTCCTGGAGG
14	bta-miR-345-5p	-1.1639041	down	-1.5862782	down	AGCACTGGACTAGGAGT
15	bta-miR-425-5p	-1.4746515	down	-1.0988923	down	TCAACGGGAGTGATCGTG
16	bta-miR-4286	-1.3730899	down	-1.4312056	down	GGTACCAGGAGTGGG
17	bta-miR-532	-1.294599	down	-1.3987232	down	ACGGTCCTACACTCAAG
18	bta-miR-208b	1.6484828	up	2.0035646	up	ACAAACCTTTTGTTCGTCTTA
19	bta-miR-499	1.5488322	up	1.9779112	up	AAACATCACTGCAAGTCTTAA
20	bta-miR-101	1.164391	up	1.9123551	up	TTCAGTTATCACAGTACTGT
21	bta-miR-148a	1.458694	up	1.0202311	up	ACAAAGTTCTGTAGTGCACT
22	bta-miR-195	1.2344453	up	1.2008775	up	TGCCAATATTTCTGTGCT
23	bta-miR-199a-5p	1.5291214	up	1.0221341	up	AACAGGTAGTCTGAACAC
24	bta-miR-99a-5p	1.0440416	up	1.2658235	up	ACAAGATCGGATCTACGG
